# The Key Enzymes in the Suberin Biosynthetic Pathway in Plants: An Update

**DOI:** 10.3390/plants11030392

**Published:** 2022-01-30

**Authors:** Gal Nomberg, Ofir Marinov, Gulab Chand Arya, Ekaterina Manasherova, Hagai Cohen

**Affiliations:** 1 Volcani Center, Department of Vegetable and Field Crops, Institute of Plant Sciences, Agricultural Research Organization (ARO), Rishon Lezion 7505101, Israel; galn@volcani.agri.gov.il (G.N.); ofirm@volcani.agri.gov.il (O.M.); arya@volcani.agri.gov.il (G.C.A.); ekaterina@volcani.agri.gov.il (E.M.); 2 Department of Plant Pathology and Microbiology, The Robert H. Smith Faculty of Agriculture, Food and Environment, The Hebrew University of Jerusalem, Rehovot 7610001, Israel

**Keywords:** plant lipophilic barriers, suberin pathway, suberin polyaliphatic domain, suberin polyphenolic domain, very long chain fatty acids (VLCFAs), core phenylpropanoid pathway

## Abstract

Suberin is a natural biopolymer found in a variety of specialized tissues, including seed coat integuments, root endodermis, tree bark, potato tuber skin and the russeted and reticulated skin of fruits. The suberin polymer consists of polyaliphatic and polyphenolic domains. The former is made of very long chain fatty acids, primary alcohols and a glycerol backbone, while the latter consists of *p*-hydroxycinnamic acid derivatives, which originate from the core phenylpropanoid pathway. In the current review, we survey the current knowledge on genes/enzymes associated with the suberin biosynthetic pathway in plants, reflecting the outcomes of considerable research efforts in the last two decades. We discuss the function of these genes/enzymes with respect to suberin aromatic and aliphatic monomer biosynthesis, suberin monomer transport, and suberin pathway regulation. We also delineate the consequences of the altered expression/accumulation of these genes/enzymes in transgenic plants.

## 1. Introduction

The colonization of land by plants commenced c. 450 million years ago with the evolution of the first streptophyte algae lineage into terrestrial angiosperms [1]. The migration from aquatic habitats to dry land forced plants to adapt to the new harsh environment in multiple ways. Recently, a comparative analysis of genes belonging to 72 plants and algae pointed to innovations and losses in the metabolic capabilities of phytohormones, as well as to the biosynthesis of structural polymers, such as cutin and suberin, over the course of land plant evolution [2]. Indeed, plants’ adoption of these two polymers enabled their outermost aerial surfaces (i.e., the cuticle layer) to be covered with lipophilic barriers. These polymers also coat some inner cellular layers of specialized tissues (i.e., the suberin lamellae). Both the cuticle and the suberin lamellae play pivotal roles in minimizing water loss through transpiration and in regulating the movement of water, solutes and gases [3,4,5]. They also act as mechanical safeguards against pathogen attacks and herbivory [6,7].

**Figure 1:**
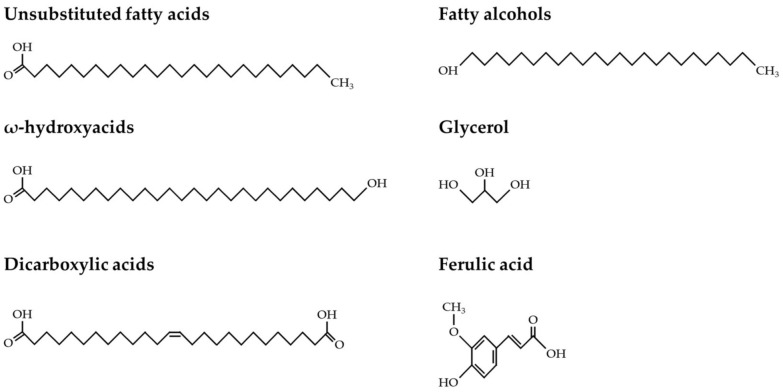
The typical monomers that build the suberin polymer in plants. Chemical formulas of constituents of the suberin polyaliphatic domain: unsubstituted fatty acids, ω-hydroxyacids, dicarboxylic acids and fatty alcohols and glycerol polymer (the major backbone of suberin). The chemical formula of ferulic acid, the most abundant monomer of the suberin polyphenolic domain, is also shown.

The cuticle layer is composed predominantly of the cutin polymer, made up of C_16_ and C_18_ ω-hydroxylated fatty acids, and is immersed in and/or coated by very long chain fatty acid (VLCFA)-derived epicuticular waxes [8]. Suberin is a more complex heteropolymer built from two distinct domains. The first is a polyaliphatic domain composed of a mixture of VLCFAs, typically with chain lengths >C_20_ (including unsubstituted fatty acids, ω-hydroxyacids and dicarboxylic acids), primary alcohols and a glycerol backbone [9,10] (Figure 1). The second is a polyphenolic domain, which is commonly made up of *p*-hydroxycinnamic acid byproducts that originate from the core phenylpropanoid pathway, with ferulic acid being the most abundant one [11,12,13] (Figure 1). 

**Figure 2:**
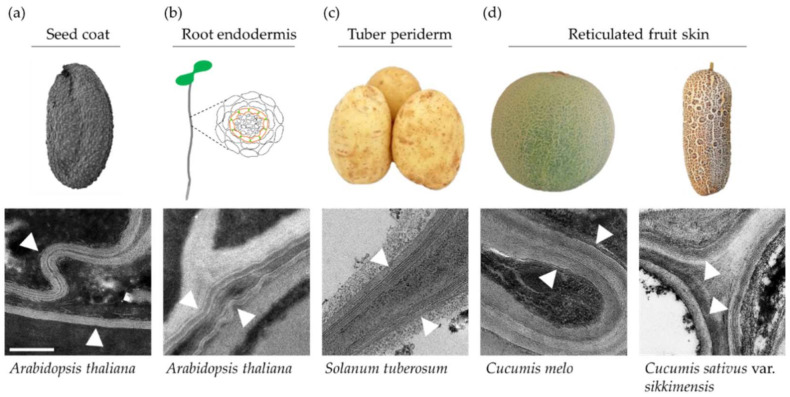
Examples of the deposition of suberin lamellae in plants. (**a**) Suberin lamellae in *Arabidopsis thaliana* seed coat integuments. (**b**) Suberin lamellae in the *Arabidopsis thaliana* root endodermis cell layer. (**c**) Suberin lamellae in the periderm tissue of potato (*Solanum tuberosum*) tuber skin. (**d**) Suberin lamellae in the reticulated fruit skin of melon (*Cucumis melo*) and cucumber (*Cucumis sativus* var. *sikkimensis*) varieties. Lower panels are transmission electron microscopy images representing suberin lamellae (indicated by white arrowheads) in different tissues. Scale bar = 200 nm.

Cutin and suberin differ not only in their chemical constituents but also in their cellular deposition sites. The cuticle layer is deposited on top of the outermost epidermal cells of all aboveground plant tissues (e.g., leaves, stems, flowers and fruit). Conversely, suberin lamellae can be found in specialized tissues, ranging from unicellular depositions in seed coat integuments (Figure 2a) and root endodermis cell layers (Figure 2b) to multicellular organs such as the bark of trees, the periderm tissues of potato tuber skin (Figure 2c), the russeted skin of apple and pear fruit species and the reticulated skin of various melon and cucumber fruit cultivars [14,15] (Figure 2). The suberin polymer is deposited in between the plasma membrane and the cell wall. Despite the fact that all suberin polymers isolated from a range of tissues were found to be principally made of similar basic monomers, their total content and composition vary greatly [16]. 

A transmission electron microscopy analysis reveals the suberin polymer to be a polylamellate ultrastructure (Figure 2). Two types of lamellae have been identified, which are distinguished by their apparent color in electron micrographs, either semitransparent or nontransparent gray. This suggests that the dissimilarities between these two types of lamellae originate from chemical attributes. Indeed, in vitro and in vivo studies have found the polyaliphatic domain to be mainly associated with the semitransparent lamellae and the polyphenolic domain to be associated with the dark lamellae [17]. It has also been demonstrated that tight spatiotemporal regulation of the biosynthetic pathways leading to the formation of these two domains is critical for the proper formation of suberin lamellae with a complete and intact ultrastructure [13]. 

The fine molecular structure of the suberin polymer has yet to be fully elucidated, most likely due to its complex polymeric nature. Correspondingly, most studies of suberin chemistry have relied on suberin extracts from different plant sources following hydrolysis and transesterification processes, which yield only monomers and/or small oligomers, not the complete chemical structure of the suberin polymer. Thus far, several hypothetical structures for the suberin polymer and its association with the cell wall have been raised (for more information, see [13,17,18]). Emerging advanced in situ and ex situ approaches may hold the potential to unravel the chemical and physical structure of the suberin polymer [16,19]. Nonetheless, the mechanisms underlying the assembly and polymerization of the building blocks of the full macromolecular structure of suberin remain to be resolved. 

In addition to the aforementioned comprehensive reviews of the macromolecular structure of the suberin polymer, several recent reviews have outlined various aspects of the suberin pathway in plants. These include the possible cellular export and trafficking mechanisms of suberin [20,21], the establishment of the suberin pathway during plant evolution [2,22], the roles played by the suberin polymer in plant responses against abiotic and biotic stresses [23,24,25] and the importance of suberin in the production of bioderived energy and materials [26]. 

In the current review, we strive to provide an updated synopsis of the major efforts made in the last two decades to unravel novel genes/enzymes operating in the suberin pathway in plants. Our main focus is on the substantial advances made in the last couple of years. For the sake of simplicity, we survey the genes/enzymes in the suberin biosynthetic pathway according to their functional roles in: the formation of polyaliphatic monomers, the formation of suberin polyphenolic monomers, the transport of suberin monomers and the regulation of the suberin pathway. All genes/enzymes discussed in this review are presented in Table 1 according to their order of appearance in the text. We further discuss the consequences of the altered expression/accumulation of these genes/enzymes in transgenic plants.

## 2. The Formation of Suberin Polyaliphatic Monomers

**Figure 3:**
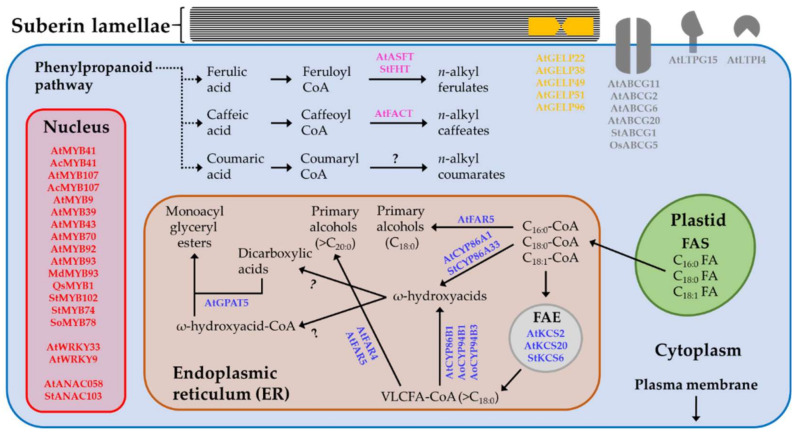
Schematic representation of the biosynthetic pathway of suberin in plants. The scheme is proposed based on previous evidence in the literature and shows all genes/enzymes listed in Table 1 according to their known enzymatic reactions. The scheme presents genes/enzymes involved in suberin polyaliphatic monomers (blue), suberin polyphenolic monomers (pink), suberin monomer polymerization (orange), suberin monomer transport (gray) and regulation of the suberin pathway (red). Dashed lines represent more than one enzymatic step, while question marks above enzymatic reactions indicate that genes/enzymes involved in these reactions are yet to be defined. Gene/enzyme abbreviations: ASFT, ALIPHATIC SUBERIN FERULOYL TRANSFERASE; CYP, CYTOCHROME P450 (FATTY ACYL ω-HYDROXYLASE); FACT, FATTY ALCOHOL:CAFFEOYL-CoA CAFFEOYL TRANSFERASE; FAR, FATTY ACYL-CoA REDUCTASE; FHT, FATTY ALCOHOL HYDROXYCINNAMOYL TRANSFERASE; GELP, GDSL-type ESTERASE/LIPASE PROTEINS; GPAT, GLYCEROL-3-PHOSPHATE ACYLTRANSFERASE; KCS, β-KETOACYL-CoA SYNTHETASE; LTP, LIPID TRANSFER PROTEIN; LTPG, GLYCOSYLPHOSPHATIDYLINOSITOL (GPI)-ANCHORED LIPID TRANSFER PROTEIN. Species abbreviations: Ac, *Actinidia chinensis*; Ao, *Avicennia officinalis*; At, *Arabidopsis thaliana*; Md, *Malus x domestica*; Os, *Oryza sativa*; Qs, *Quercus suber*; So, *Saccharum offcinarum*; St, *Solanum tuberosum*.

Suberin biosynthesis relies on the synthesis of C_16:0_, C_18:0_ and C_18:1_ fatty acids in plastids by the fatty acid synthase (FAS) complex (Figure 3). Following the addition of CoA groups, C_16:0_-CoA, C_18:0_-CoA and C_18:1_-CoA can be transported into the endoplasmic reticulum (ER), where they are elongated into VLCFAs via the fatty acid elongation (FAE) complex (Figure 3).

The first committed step in VLCFA biosynthesis is the condensation of C(2) units to acyl-CoA via the activity of *β*-KETOACYL-CoA SYNTHETASE (KCS) enzymes (Figure 3). Two early studies isolated *KCS2/DAISY* and *KCS20* and demonstrated that they encode fatty acid elongase condensing enzymes essential for the biosynthesis of aliphatic suberin [27,63]. The authors showed that KCS2 localizes to roots and the chalaza–micropyle region of seeds [16] and that both encoded enzymes are functionally redundant but differentially controlled under osmotic stress [27]. Further analyses showed that roots of the Arabidopsis *atkcs2/daisy* mutant exhibited retarded growth and that their suberin composition, compared to that of the wild type, was depleted in C_22_ and C_22_ VLCFAs but enriched in C_16_, C_18_ and C_20_ derivatives [63]. Correspondingly, the roots of the Arabidopsis *atkcs20kcs2* double-mutant were growth-retarded and featured an abnormal endodermis suberin lamella structure. Chemical analysis of these roots revealed a significant reduction in C_22_ and C_24_ VLCFAs levels but an overaccumulation of C_20_ VLCFA derivatives in aliphatic suberin [27]. In addition to these Arabidopsis KCSs, potato (*Solanum tuberosum*) KCS6 was suggested to be involved in the formation of suberin monomers with chain lengths >C_28_, as the silencing of this gene in the tuber skin periderm tissue explicitly reduced the levels of suberin-related VLCFAs with lengths above C_28_ [28]. 

Suberin-related VLCFAs are further oxygenated by members of the CYTOCHROME P450 OXIDASE protein family (Figure 3). Using reverse genetics, Höfer et al. [29] isolated and characterized CYP86A1/HORST as a key enzyme in aliphatic suberin biosynthesis in Arabidopsis roots. Notably, *atcyp86a1/horst* mutant roots synthesized significantly lower levels of ω-hydroxyacids, particularly those with chain lengths shorter than C_20_ [29]. Similar approaches enabled the identification of CYP86B1/RALPH as a key oxidase in the biosynthesis of suberin ω-hydroxyacids and α,ω-dicarboxylic acids in Arabidopsis roots and seeds. Chemical assays of roots and seeds belonging to a subset of *atcyp86B1/ralph* mutants, including knockout lines and RNA interference-silenced lines, showed a strong reduction in the levels of C_22_ and C_24_ ω-hydroxyacids and α,ω-dicarboxylic acids [30,31]. CYP86A33 from potato has also been suggested to be an oxidase involved in suberin synthesis in tuber skin periderm tissue. CYP86A33-RNAi lines produced tubers marked by a 60% reduction in aliphatic suberin loads, with a particular decrease in C_18:1_ ω-hydroxy and α,ω-diacid levels, leading to a distorted, much thinner suberin lamella ultrastructure [32]. Recently, CYP94B1 from the salt secretor mangrove *Avicennia officinalis* and its putative ortholog in Arabidopsis were shown to control root suberin biosynthesis. Heterologous expression of *AoCYP94B1* in the Arabidopsis *atcyp94b1* mutant and in wild-type rice (*Oryza sativa*) resulted in a higher accumulation of suberin content, eventually leading to increased tolerance against salt stress [33]. Similar approaches enabled the identification of CYP94B3 as an oxidase involved in the suberin pathway. The reduced suberin content detected in the roots of the corresponding Arabidopsis *atcyp94b3* mutant was rescued when it expressed AoCYP94B3 [34]. 

Another archetypal class of suberin monomers is composed of primary alcohols. The formation of these components is executed by the reduction of carboxyl groups via the activity of FATTY ACYL-CoA REDUCTASE (FAR) enzymes (Figure 3). Using a subset of transgenic lines expressing the β-glucoronidase reporter gene under the control of promoters belonging to the Arabidopsis family of eight FARs, Domergue et al. [35] showed that the FAR1, FAR4 and FAR5 triad is expressed in root endodermal cells coinciding with known sites of suberin deposition. Arabidopsis mutant lines of FAR1, FAR4 and FAR5 displayed altered suberin compositions in roots and seed coats, specifically a respective reduction in C_22_, C_20_ and C_18_ primary alcohols. Heterologous expression of the three FARs in yeast established their activity as alcohol-forming enzymes with distinct but overlapping chain-length specificities ranging from C_18_ to C_24_ [35]. A subsequent study revealed that the downregulation of these three FARs in triple-*far* mutants reduced the levels of suberin primary alcohols by 70% to 80% in the polymeric and nonpolymeric fractions of roots of tissue-culture-grown plants. They also featured a reduction in suberin-associated root waxes of seven-week-old soil-grown plants and a decrease in the seed coat suberin polymer [64].

Glycerol is considered the backbone of the suberin polymer. Beisson et al. [36] showed that Arabidopsis GLYCEROL-3-PHOSPHATE ACYLTRANSFERASE5 (GPAT5) produces monoacylglyceryl esters by attaching glycerol to ω-hydroxyacyl-CoA and α,ω-dicarboxyacyl-CoA components (Figure 3). Compared to wild-type roots, *atgpat5* mutant roots had 50% less aliphatic suberin, and their seed coats had seven-fold lower suberin-related dicarboxylic acid and ω-hydroxyacid contents [36]. Subsequent studies showed that the overexpression of GPAT5 can modify the composition of the cutin polymer by introducing newly formed C_20_ and C_22_ ω-hydroxyacids and dicarboxylic acids, typical of suberin [65]. Interestingly, GPAT4, 6 and 8, which are associated with cutin biosynthesis, showed strict preference for C_16_ and C_18:1_ ω-oxidized acyl-CoA components over ones with longer chains. GPAT5, however, acts on a wide range of long-chain ω-oxidized and/or unsubstituted acyl-CoA components [66]. This body of evidence suggests that members of the GPAT family have undergone specialization during evolution. Indeed, phylogenetic assays have demonstrated that GPATs associate with cutin, which arose early during land plant evolution, forming a different cluster from that of GPATs associated with suberin, which appeared much later in evolution [66]. Another enzyme with possible roles in the biosynthesis of suberin aliphatics is ENHANCED SUBERIN1 (ESB1), as Arabidopsis *atesb1* mutants display a two-fold increase in root suberin content [37]. This protein has been shown to be part of the machinery required for the formation of Casparian strips in root endodermis that are made of lignin without suberin [67]. Therefore, its direct roles in suberin metabolism remain to be fully elucidated. 

Recently, a subset of five auxin-mediated GDSL-type esterase/lipase proteins (GELPs), GELP22, GELP38, GELP49, GELP51 and GELP96, were proposed to play a role in suberin polymerization in Arabidopsis roots (Figure 3). The expression of these five genes strongly correlated with endodermal suberization patterns. Moreover, the simultaneous knockout of these five genes in Arabidopsis *gelp^quint^* mutant lines led to an 85% decrease in the total suberin monomer content [38]. Remarkably, the authors also isolated an additional subset of auxin-inducible GELPs with the capacity to degrade endodermal suberin when required, for example, during lateral root emergence [38].

## 3. The Formation of Suberin Polyphenolic Monomers

The suberin polyphenolic constituent of the suberin polymer heavily depends on the supply of precursors from the core phenylpropanoid pathway. Indeed, transesterification of suberin from different samples resulted in the release of diverse aliphatic monomers, along with *p*-hydroxycinnamic acids such as cinnamic, coumaric and caffeic acids, with ferulic acid being the predominant one [8,13,68,69]. The transfer of ferulic acid to the aliphatic suberin building blocks is executed by ALIPHATIC SUBERIN FERULOYL TRANSFERASE (ASFT), a member of the BAHD family of acetyltransferases (Figure 3). Two parallel studies have shown that *ASFT* is expressed in seed coat and root endodermis where suberization occurs [31,39]. Further, Arabidopsis knockout *atasft* mutants are almost completely devoid of ferulic acid in their polyphenolic domain, accompanied by substantial effects on the aliphatic suberin monomer levels [31,39]. Despite these chemical differences, the contents of suberin-related alkyl hydroxycinnamate ester waxes were genuine in *atasft* mutants and similar to those of the wild type. An additional member of the BAHD family, namely, FATTY ALCOHOL:CAFFEOYL-CoA CAFFEOYL TRANSFERASE (FACT), was shown to catalyze the synthesis of alkyl hydroxycinnamate ester waxes, particularly alkyl caffeate esters (Figure 3). Thus, FACT acts as an incorporator of caffeate into the suberin polymer [40]. Another feruloyl transferase, FATTY ALCOHOL HYDROXYCINNAMOYL TRANSFERASE (FHT), was reported in potato (Figure 3). The generation of FHT-RNAi potato plants produced tubers depleted in skin periderm suberin and suberin-associated waxes. While an intact suberin lamellae ultrastructure was observed in FHT-RNAi tubers, they exhibited severe morphological aberrations. In vitro evaluations of FHT’s enzymatic aptitudes verified its ability to conjugate ferulic acid with ω-hydroxyacids and fatty alcohols [41]. The importance of phenylpropanoids, primarily ferulic acid, for suberin biosynthesis was recently highlighted by showing that endodermal suberin deposition is disrupted by the inhibition of phenylpropanoid synthesis, which could be restored by adding ferulic acid [70].

## 4. Suberin Monomer Transport

Given that suberin is comprised of both insoluble and soluble monomers, it is presumed that the export of suberin building blocks from their sites of biosynthesis to their deposition sites is a relatively complex process. In fact, the transport mechanisms underlying these processes are the most poorly understood aspect of the suberin pathway. 

Previous in silico and in vivo assays positioned many of the suberin monomer’s biosynthetic enzymes in the endoplasmic reticulum (ER). Thus, it is predicted that the synthesis and modifications of aliphatic monomers occur in the ER. The core phenylpropanoid pathway that provides *p*-hydroxycinnamic precursors for suberin polyphenolic components, however, is known to take place in the cytosol. Pollard et al. [8] projected several mechanisms by which suberin monomers and/or oligomers and/or a complete polymer might be exported from the ER to their deposition sites in between the plasma membrane (PM) and the cell wall: (i) ER domains anchored to the PM facilitate transport of suberin components, (ii) cytosolic carriers transport soluble proteins that contain suberin components through the cytosolic space to the PM, (iii) oleophilic droplets that contain suberin components bud from the ER membrane and travel through the cytosol to the PM and (iv) Golgi-mediated secretion occurs via specialized vesicles that contain suberin components [8]. In agreement with the latter mechanism, an old report inferred that ER-derived ribosome-bearing vesicles carry suberin aliphatic precursors towards the PM and cell walls of a developing pigment strand of rice (*Oryza sativa*) [71]. 

Regardless of which of these transport models will be revealed as the correct mode of suberin monomer transport, the apparent involvement of transporters, such as those belonging to the half-size ATP-BINDING CASSETTE (ABCG) and/or LIPID TRANSFER PROTEIN (LTP) superfamilies, is expected once suberin components reach the PM. Arabidopsis DSO/ABCG11 was the first transporter suggested to play a role in suberin monomer export. This was based on the observation that repressing DSO/ABCG11 expression in *atdso/atabcg11* mutants resulted in lower suberin content and suberin-related gene transcripts (Figure 3). Despite these changes, ABCG11 was primarily associated with cutin metabolism in aboveground reproductive organs, while its possible role in suberin monomer transport remains to be fully validated [42]. An additional three Arabidopsis ABCG transporters, ABCG2, 6 and 20, were associated with suberin metabolism in roots and seed coats (Figure 3). Chemical profiling of roots and seed coats produced by the triple-mutant *atbcg2atabcg6atabcg20* pointed to lower suberin content along with a distorted structure of their suberin lamellae and increased permeability [43]. Later, *awake-1*, an allele of ABCG20, was shown to play essential roles in the transport of fatty acids during suberin deposition in Arabidopsis seeds [44]. Moreover, potato ABCG1 was highlighted as a key transporter of suberin monomers during periderm formation (Figure 3). ABCG1-RNAi-silenced potato plants exhibited major morphological perturbations of both the tubers and root exodermis, reduced suberin levels and malformed suberized cell layers [45]. A subsequent study examined the contribution of Arabidopsis ABCG1 to suberin formation in roots. Metabolite profiling of the *atabcg1* mutant roots revealed reduced suberin content, especially in VLCFAs, primary alcohols and dicarboxylic acids. Direct functional assays of purified AtABCG1 revealed a preference for C_24_-C_30_ fatty acids and C_26_-C_30_ fatty alcohols, which corroborated the chemical changes detected in the root suberin of the *atabcg1* mutant [72]. Lastly, the RCN1/ABCG5 of rice was shown to participate in the hypodermal suberization of roots (Figure 3). The authors isolated a rice mutant line named *reduced culm number1* (*rcn1*), which failed to develop long roots, apparently due to lower levels of C_28_ and C_30_ fatty acids and ω-hydroxyacids [46].

LTPs were previously suggested to play roles in the transfer of monomers required for the establishment of cutin and epicuticular waxes (reviewed by [73]). However, evidence of a possible role for these proteins in suberin monomer transport is scarce. Thus far, Arabidopsis LTPI4 was suggested to participate in suberin production of Arabidopsis crown gall tumors, which develop upon infection with virulent Agrobacterium strains (Figure 3). These structures are enclosed by a suberized periderm. A study showed that *atltpi4* mutant crown galls accumulated much less suberin, attributed to decreased synthesis of C_18_ VLCFAs. The ability of this transporter to act on VLCFAs typical to suberin was also demonstrated by expressing the protein in epidermis cells, which resulted in a significant rise in the levels of C_24_ and C_26_ VLCFAs [47]. Finally, Arabidopsis *LTPG15*, encoding GLYCOSYLPHOSPHATIDYLINOSITOL (GPI)-ANCHORED LIPID TRANSFER PROTEIN, was shown to be expressed in suberin-producing tissues of the root endodermis and seed coat (Figure 3). Seeds produced by the corresponding *atltpg15* mutant synthesized lower levels of C_20_-C_24_ fatty acids, C_20_ and C_22_ primary alcohols, C_22_ and C_24_ ω-hydroxyacids and C_20_ and C_22_ dicarboxylic acids [48].

## 5. Regulation of the Suberin Pathway

Insights into the regulation of the suberin pathway were obtained only in the last decade. The first transcription factor identified as a positive regulator of suberin was Arabidopsis MYB41 (Figure 3). It was shown that MYB41 can activate the steps necessary for the synthesis of aliphatic suberin and for the deposition of a cell-wall-associated suberin-like lamellae in both *Arabidopsis thaliana* and *Nicotiana benthamiana*, seemingly by inducing the expression of suberin gene transcripts [49]. Other members of the MYB family were shown to regulate suberin deposition in multiple species and tissues. Using multispecies coexpression analyses, Lashbrooke et al. [52] isolated Arabidopsis MYB107 and MYB9 homologs, which appeared to synchronize the transcriptional induction of aliphatic and aromatic monomer biosynthesis and transport and suberin polymerization in the outer integument layer (Figure 3). The corresponding *atmyb1097* and *atmyb9* mutant seeds displayed significant reductions in suberin content and altered levels of other seed-coat-associated metabolites [52]. Later, MYB107 was shown to induce the expression of suberin genes by physically interacting with regulatory elements in their promoters [53]. Interestingly, the putative orthologs of MYB107 and MYB41 in kiwifruit (*Actinidia chinensis* Planch cv. Xuxiang) were shown to interact with the kiwifruit *CYP86A1* promoter to activate the expression of its corresponding gene. *Nicotiana benthamiana* leaves expressing these two kiwifruit MYBs exhibited induced expression of suberin aliphatic genes and the accumulation of typical suberin components such as ω-hydroxyacids, dicarboxylic acids and primary alcohols [50]. A distinct MYB107, MYB9 and MYB39 (SUBERMAN) phylogenetic clade was shown to regulate endodermal suberization in Arabidopsis roots (Figure 3). Similar to MYB41, transient expression of Arabidopsis SUBERMAN in *Nicotiana benthamiana* leaves induced the accumulation of suberin and the deposition of a suberin-like lamellar structure [54]. SUBERMAN expression was confined to the root endodermis cell layer, and when overexpressed in transgenic Arabidopsis, it led to the ectopic deposition of suberin lamellae even in the root cortex and epidermis cell layers. Expectedly, the corresponding Arabidopsis *atsub* mutant roots accumulated less suberin content compared to wild-type roots [54]. More recently, Shukla et al. [51] showed that MYB41, MYB53, MYB92 and MYB93 all promote endodermal suberization in Arabidopsis roots (Figure 3). The authors mutated these four MYBs simultaneously through genome-editing tools, which led to a dramatic reduction in suberin formation in response to both developmental and environmental signals [51]. As in the cases of MYB41 and MYB39, when MYB92 was transiently expressed in *Nicotinana benthamiana* leaves, it affected lipid homeostasis and yielded a 50-fold increase in suberin deposition [74]. Apart from Arabidopsis, several MYBs from other species have been suggested to play a regulatory role in suberin biosynthesis. For example, cork oak (*Quercus suber*) MYB1 was shown to target genes involved in both suberin and lignin biosynthesis, transport and assembly, which are tightly associated with secondary growth and cork development [55] (Figure 3). MYB93 from apple (*Malus x domestica*) regulates suberin deposition during the formation of russeting on top of fruit skin during development [56]. Recently, potato (*Solanum tuberosum*) *MYB102* and *MYB74* genes were suggested as important regulators of wound suberization processes in potato tubers (Figure 3). Again, transient expression of these two potato MYBs in *Nicotiana benthamiana* leaves induced the deposition of a suberin-like lamellar ultrastructure and monomers characteristic of polyaliphatic suberin [57]. Arabidopsis MYB70 was recently proposed as a negative regulator of suberin biosynthesis in roots, as transcriptome assays showed reduced expression levels of suberin-related genes in MYB70-overexpressing plants that also exhibited a striking reduction in almost all root suberin aliphatic constituents (Figure 3). A yeast-1-hybrid (Y1H) assay, electrophoretic mobility shift assay (EMSA) and chromatin immunoprecipitation coupled with quantitative PCR (ChIP-qPCR) analysis validated that MYB70 physically interacts with elements in the GPAT5 gene promoter. Interestingly, the suberin content in the corresponding *atmyb70* mutants was not affected [58]. Lastly, through a screen for genes upregulated in sugarcane (*Saccharum offcinarum*) internodes undergoing suberization during culm development or due to wounding, Figueiredo et al. [59] identified MYB78 as a candidate regulator of suberin metabolism (Figure 3). The authors demonstrated that MYB78 induces suberin deposition in *Nicotiana benthamiana* leaves by activating the expression of suberin biosynthetic genes [59]. This is important evidence, as, to our knowledge, it is the first identified regulator of suberin in C4 grasses and monocots. 

Transcriptional regulators of the suberin pathway were also identified in the WRKY and ANAC (NAM/ATAF/CUC) protein facilities. For instance, Arabidopsis WRKY33 was recently identified as an upstream regulator of CYP94B1 oxidase, associated with suberin biosynthesis (Figure 3). In agreement, the corresponding atwrky33 mutant roots accumulated less suberin and were more sensitive to salt, and these phenotypes were rescued upon the overexpression of CYP94B1 in the background of this mutant [33]. By means of comparable methods, the same research group further identified Arabidopsis WRKY9 as an upstream regulator of two other suberin oxidases, CYP94B3 and CYP86B1 (Figure 3). In addition, *atwrky9* mutant roots showed repressed gene expression of CYP94B3 and CYP86B1 and had less suberin content [34]. Lastly, Arabidopsis ANAC046 was proposed as a transcriptional activator of suberin (Figure 3). It was demonstrated that its expression is confined to the suberin-accumulating tissues of the root endodermis and periderm, and its overexpression in transgenic Arabidopsis resulted in a two-fold increase in root suberin [60]. 

Unlike all the aforementioned transcriptional factors that positively regulate suberin biosynthesis, potato ANAC103 seems to negatively regulate it (Figure 3). Silencing ANAC103 in the potato periderm was accompanied by increased amounts of suberin and wax components, indicating a negative effect on these pathways [61]. Arabidopsis ANAC058, an ortholog of potato ANAC103, was shown to be exclusively expressed in the Arabidopsis root endodermis and to participate in endodermal suberization. A subset of knockout and knockdown *atanac058* mutants displayed delayed root suberization, while the overexpression of ANAC058 caused ectopic deposition of suberin in roots [62].

## 6. Conclusions and Future Prospective

The extensive study, over several decades, of the pathways underlying the formation of the cutin polymer, the major constituent of the cuticle, has led to the characterization of many genes and components involved in these processes. However, the first enzymes involved in suberin biosynthesis and deposition in plants were identified only in the last two decades, with many missing parts still remaining in the puzzle. In contrast to the cuticle, which covers all aerial parts of plants, the suberin lamella is hidden within the cells of specialized tissues, making it harder to investigate. 

Several key aspects of the suberin pathway remain elusive, particularly those involved in the polymerization of the suberin building blocks, the means of transport of suberin monomers to their deposition sites and the general chemical structure of the suberin polymer [5]. Nonetheless, it is expected that the recently emerged ‘omics’-based technologies will assist in further elucidating the mechanisms underlying these processes [4]. In conclusion, future genomics studies accompanied by chemical structural examinations are required in order to tackle the unresolved aspects of suberin metabolism. These are crucial for a deeper understanding of suberin biosynthesis and regulation in plants, as well as for the production of suberin-based byproducts of renewable and sustainable plant origins, which are emerging as promising industrial and applicative biotechnological agents.

## Figures and Tables

**Table 1:** List of genes/enzymes that were functionally characterized in the suberin pathway. Genes/enzymes are listed according to their order of appearance in the main text.

Enzyme Function	Species	Enzyme Name	Reference(s)
*β*-KETOACYL-CoA SYNTHETASE	*Arabidopsis thaliana*	AtKCS2/DAISY	[27]
	*Arabidopsis thaliana*	AtKCS20	[27]
	*Solanum tuberosum*	StKCS6	[28]
CYTOCHROME P450 (FATTY ACYL ω-HYDROXYLASE)	*Arabidopsis thaliana*	AtCYP86A1/HORST	[29]
	*Arabidopsis thaliana*	AtCYP86B1/RALPH	[30,31]
	*Solanum tuberosum*	StCYP86A33	[32]
	*Avicennia officinalis*	AoCYP94B1	[33]
	*Avicennia officinalis*	AoCYP94B3	[34]
FATTY ACYL-CoA REDUCTASE	*Arabidopsis thaliana*	AtFAR1	[35]
	*Arabidopsis thaliana*	AtFAR4	[35]
	*Arabidopsis thaliana*	AtFAR5	[35]
GLYCEROL-3-PHOSPHATE ACYLTRANSFERASE	*Arabidopsis thaliana*	AtGPAT5	[36]
DIRIGENT-like PROTEIN	*Arabidopsis thaliana*	AtESB1	[37]
GDSL-type ESTERASE/LIPASE PROTEINS	*Arabidopsis thaliana*	AtGELP22	[38]
	*Arabidopsis thaliana*	AtGELP38	[38]
	*Arabidopsis thaliana*	AtGELP49	[38]
	*Arabidopsis thaliana*	AtGELP51	[38]
	*Arabidopsis thaliana*	AtGELP96	[38]
FERULOYL TRANSFERASE	*Arabidopsis thaliana*	AtASFT	[30,39]
	*Arabidopsis thaliana*	AtFACT	[40]
	*Solanum tuberosum*	StFHT	[41]
ATP-BINDING CASSETTE	*Arabidopsis thaliana*	AtDSO/ABCG11	[42]
	*Arabidopsis thaliana*	AtABCG2	[43]
	*Arabidopsis thaliana*	AtABCG6	[43]
	*Arabidopsis thaliana*	AtABCG20	[43,44]
	*Solanum tuberosum*	StABCG1	[45]
	*Oryza sativa*	OsRCN1/ABCG5	[46]
LIPID TRANSFER PROTEIN	*Arabidopsis thaliana*	AtLTPI4	[47]
	*Arabidopsis thaliana*	AtLTPG15	[48]
MYB proteins	*Arabidopsis thaliana /* *Actinidia chinensis*	AtMYB41	[49,50,51]
	*Arabidopsis thaliana /* *Actinidia chinensis*	AtMYB107	[50,52,53]
	*Arabidopsis thaliana*	AtMYB9	[52]
	*Arabidopsis thaliana*	AtMYB39/SUBERMAN	[54]
	*Arabidopsis thaliana*	AtMYB53	[51]
	*Arabidopsis thaliana*	AtMYB92	[51]
	*Arabidopsis thaliana*	AtMYB93	[51]
	*Quercus suber*	QsMYB1	[55]
	*Malus x domestica*	MdMYB93	[56]
	*Solanum tuberosum*	StMYB102	[57]
	*Solanum tuberosum*	StMYB74	[57]
	*Arabidopsis thaliana*	AtMYB70	[58]
	*Saccharum offcinarum*	SoMYB78	[59]
WRKY proteins	*Arabidopsis thaliana*	AtWRKY33	[33]
	*Arabidopsis thaliana*	AtWRKY9	[34]
ANAC proteins	*Arabidopsis thaliana*	AtANAC046	[60]
	*Solanum tuberosum*	StANAC103	[61]
	*Arabidopsis thaliana*	AtANAC058	[62]

## Data Availability

Not applicable.
